# Tofacitinib: a promising agent for the treatment of persistent rashes in juvenile dermatomyositis

**DOI:** 10.1007/s12519-025-00901-x

**Published:** 2025-04-14

**Authors:** Zhao-Yu Chen, Tian-Nan Zhang, Ji Li, Zhen-Jie Zhang, Hong-Mei Song

**Affiliations:** 1 Department of Pediatrics, Peking Union Medical College Hospital, Chinese Academy of Medical Sciences and Peking Union Medical College, No. 1 Shuaifuyuan, Dongcheng, Beijing, 100730 China; 2 Department of Pediatrics, Tongji Hospital, Tongji Medical College, Huazhong University of Science and Technology, Wuhan, China

Juvenile dermatomyositis (JDM) is a chronic pediatric autoimmune disease characterized by proximal muscle weakness and distinctive skin manifestations [1, 2]. The first-line treatment for JDM involves the combination of glucocorticoids with either methotrexate (MTX) or cyclosporine [3, 4]. For severe and refractory juvenile dermatomyositis, options such as tacrolimus, mycophenolate mofetil (MMF), cyclophosphamide, rituximab, tumor necrosis factor-α inhibitors, and intravenous immunoglobulin (IVIG) may be considered [4–7]. Recently, several studies have reported successful management with Janus kinase (JAK) inhibitor in patients with severe or refractory JDM [8–10]. However, in clinical practice, although the standard treatment regimen, glucocorticoids plus MTX, effectively manages myositis in patients with JDM, it often fails to adequately address persistent rashes [11]. Although a previous survey conducted by the Childhood Arthritis and Rheumatology Research Alliance identified IVIG, MMF, and cyclosporine as typical treatments for JDM patients with persistent skin rash even after complete resolution of muscle involvement, there is still a lack of support from prospective data collection and statistical methods that account for nonrandom treatment assignment [11]. To date, no appropriate treatment regimen for such persistent rashes in JDM patients has been reported, making these cases particularly challenging. The question arises whether JAK inhibitors would exhibit similarly beneficial therapeutic effects in JDM patients with controlled myositis activity but persistent rashes. In this report, we detail our therapeutic experience with tofacitinib (TOF) in eight patients with refractory JDM. Although these patients had controlled myositis, they continued to suffer from persistent rashes, which were relieved significantly following the introduction of TOF.

These patients were diagnosed and classified according to the Bohan and Peter criteria [12, 13], with inactive myositis for more than six months while continuing to suffer from persistent rashes. Prior to initiating TOF treatment, all patients had undergone the first-line regimen of glucocorticoids combined with MTX and had attempted to manage their rashes with treatments such as oral hydroxychloroquine, thalidomide, or topical tacrolimus without success. TOF was administered at doses of 2.5 mg twice daily for patients weighing < 25 kg and 5 mg twice daily for those weighing ≥ 25 kg [9]. Patients maintained their original steroid treatment, which was gradually tapered according to a predefined plan, while other immunosuppressants were discontinued. The exclusion criteria included patients with severe juvenile dermatomyositis, those who had previously received tofacitinib treatment, and individuals who did not adhere to regular follow-up appointments. Written informed consent was obtained from the guardians of all patients before treatment commenced.

The demographic and clinical characteristics of the eight patients are presented in Table 1. Among the eight patients, 87.5% (7/8) were female, with an average onset age of 6.4 ± 3.2 years. The average disease duration before TOF treatment was 3.4 years, ranging from 2.9 to 8.4 years. The levels of creatine kinase (CK) and lactate dehydrogenase (LDH) at the start of TOF treatment were 93.4 ± 50 U/L and 255 ± 46.1 U/L, respectively. Four patients tested negative for myositis-specific autoantibodies (MSAs), two were positive for anti-nuclear matrix protein 2 (NXP2), one was positive for the transcriptional intermediary factor 1 (TIF1γ) antibody, and one was positive for the threonyl transport RNA (tRNA) synthetase (PL-7) antibody. Previously, seven patients had been treated with steroids, eight with MTX, four with hydroxychloroquine, one with thalidomide, and one with tacrolimus cream. The average follow-up duration was 3.0 years, ranging from 1.5 to 4.7 years. All patients showed alleviations in rashes, with 25% (2/8) achieving complete resolution. Notably, improvements began within 1–2 months of initiating TOF treatment and were significant by six months. The disease activity score (DAS) of the skin significantly decreased from an average of 5.8 (range 3.0–7.0) to 1.5 (range 0.0–2.0) (*P* < 0.001). As of the last follow-up in August 2024, 71.4% (5/7) of the patients had successfully discontinued glucocorticoids. Currently, two patients have relapses; one experienced rash recurrence eight months after TOF treatment but improved after treatment with IVIG, baricitinib, and upadacitinib. The other patient, who had been under long-term follow-up and stable disease control, discontinued TOF under medical supervision after being on treatment for three years. Four months after TOF stopped, the patient experienced rash recurrence. This patient’s symptoms were alleviated upon reintroduction of the TOF. Another patient, who experienced exacerbated calcification, discontinued TOF after one year and two months of treatment, was switched to treatment with MMF in combination with tumor necrosis factor-α inhibitors. Throughout our observation period, there were no instances of cytomegalovirus, Epstein–Barr virus, varicella-zoster virus, or tuberculosis infections, and no thrombotic events were noted, which is consistent with findings from previous studies [10, 14].

**Table 1:** Characteristics of the patients

Patient number	Sex	Age at onset (y)	MSA	Duration before TOF treatment (y)	Main feature	Other feature	CK (U/L)	LDH (U/L)	DAS-S before TOF treatment	DAS-S after TOF treatment	Drugs received before TOF treatment
1	F	6.3	N	3.5	Rash	No	107	249	6	2	Prednison, MTX, HCQ
2	F	5.6	NXP2	1.4	Rash	No	24	295	7	2	Prednison, MTX, Topical Tacrolimus
3	F	5.0	PL-7	8.1	Rash	No	108	287	5	2	Prednison, MTX, Thalidomide
4	F	6.8	N	7.0	Rash	No	104	179	8	2	MTX, HCQ
5	F	3.0	N	2.5	Rash	No	63	245	7	2	Prednison, MTX
6	F	3.5	TIF1γ	3.6	Rash	No	64	245	4	0	Prednison, MTX, HCQ
7	M	13.3	NXP2	1.5	Rash, skin ulcer	No	177	248	6	2	Prednison, MTX, HCQ
8	F	7.7	N	3.9	Rash	Calcinosis	100	214	3	2	Prednison, MTX, Pamidronate

*F* female, *M* male, *JDM* juvenile dermatomyositis, *MSA* myositis-specific autoantibody, *N* negative, *NXP2* nuclear matrix protein 2, *PL-7* threonyl-tRNA synthetase, *tRNA* transport RNA, *TIF1γ* transcriptional intermediary factor-1 gamma, *CK* creatinine kinase, *LDH* lactate dehydrogenase, *DAS-S* Disease Activity Score (DAS) of skin, *TOF* tofacitinib, *MTX* methotrexate, *HCQ* hydroxychloroquine

This study summarized the use of TOF in JDM patients with persistent skin rash following complete resolution of muscle involvement. TOF also facilitates the reduction or cessation of glucocorticoid use in these patients without significant adverse effects.

Previously, despite the concurrent use of prednisolone and MTX as first-line treatments and their beneficial effects, nearly 40% to 60% of JDM patients continue to exhibit active disease manifestations [15]. Research has shown that the type I interferon (IFN) signaling pathway is a key factor in the etiology of JDM [16, 17]. JAK inhibitors have been demonstrated to ameliorate musculoskeletal and cutaneous manifestations in JDM patients by modulating the IFN signaling pathway [18].

TOF has been reported to improve outcomes in JDM patients who are refractory to two or more disease-modifying anti-rheumatic drugs (DMARDs) or high-dose steroids [9, 14]. Although numerous studies have established the efficacy of TOF in JDM [1, 9, 14, 19, 20], our investigation represents a pioneering effort in consolidating the therapeutic effects of TOF specifically in JDM patients with persistent skin rashes. These patients had received first-line therapy consisting of glucocorticoids combined with MTX, achieving inactive myositis for more than six months while continuing to suffer from persistent rashes. Our study suggests that TOF may be an ideal option for managing refractory JDM rashes, with good overall safety.

TOF has also been reported to improve calcinosis and prevent the recurrence of JDM [20, 21]. However, our study yielded somewhat different findings. Among the eight patients who initiated TOF treatment, none had calcification at baseline. During disease progression, calcinosis emerged in one patient. Moreover, one patient experienced rash recurrence eight months after initiating TOF treatment. Although current studies demonstrate the favorable therapeutic efficacy of TOF in treating JDM, specific data regarding its potential to prevent JDM relapses and calcinosis are still lacking. Consequently, additional prospective studies are warranted to ascertain whether TOF therapy for JDM can effectively prevent disease recurrence.

## Data Availability

Data are available on reasonable request.
